# Antimicrobial peptides in human synovial membrane as (low-grade) periprosthetic joint infection biomarkers

**DOI:** 10.1186/s40001-020-00434-1

**Published:** 2020-08-17

**Authors:** Ingo J. Banke, Niko Stade, Peter M. Prodinger, Jutta Tübel, Alexander Hapfelmeier, Rüdiger von Eisenhart-Rothe, Martijn van Griensven, Hans Gollwitzer, Rainer Burgkart

**Affiliations:** 1 Clinic of Orthopedics and Sports Orthopedics, Klinikum rechts der Isar, Technical University of Munich, Ismaninger Strasse 22, 81675 Munich, Germany; 2 Institute of Medical Informatics, Statistics and Epidemiology, Klinikum rechts der Isar, Technical University of Munich, Ismaninger Strasse 22, 81675 Munich, Germany; 3 Department of Experimental Trauma Surgery, Klinikum rechts der Isar, Technical University of Munich, Ismaninger Strasse 22, 81675 Munich, Germany; 4 Department cBITE, MERLN Institute, Maastricht University, Maastricht, The Netherlands; 5 ECOM–Excellent Center of Medicine, Arabellastrasse 17, 81925 Munich, Germany

**Keywords:** Joint replacement, Arthroplasty, Periprosthetic infection, Biomarker, Synovial membrane, Histological diagnosis, Antimicrobial peptide (AMP), Human beta-defensin (HBD), Cathelicidin (LL-37)

## Abstract

**Background:**

Safe diagnosis of periprosthetic joint infection (PJI) is of utmost importance for successful exchange arthroplasty. However, current diagnostic tools show insufficient accuracy in the clinically common and challenging chronic low-grade infections. To close this diagnostic gap, reliable (bio)markers display the most promising candidates. Antimicrobial peptides (AMPs) are part of the innate immune response towards microbial growth. Recently we could show significant intraarticular levels of human cathelicidin LL-37 and β-defensin-3 (HBD-3) with high diagnostic accuracy in PJI synovial fluid. Consequently, these promising biomarkers were evaluated in PJI synovial membrane and synoviocytes, which may significantly facilitate histological diagnosis of PJI to improve outcome of septic joint replacement.

**Methods:**

In this prospective single-center controlled clinical study (diagnostic level II), consecutive patients with total hip (THR) and knee (TKR) replacements were included undergoing primary arthroplasty (*n* = 8), surgical revision due to aseptic loosening (*n* = 9) and septic arthroplasty with coagulase-negative staphylococci (*n* = 8) according to the criteria of the Musculoskeletal Infection Society (MSIS). Semiquantitative immunohistochemical (IHC) analysis of LL-37, HBD-3 and HBD-2 in synovial membrane and isolated synoviocytes based on Total Allred Score (TS) and Immunoreactive Remmele and Stegner score (IRS) was performed. For statistical analysis, SPSS 26.0/R3.6.3 (*p* < 0.05) was used.

**Results:**

The AMPs LL-37 and HBD-3 were significantly elevated (up to 20×) in synovial membranes from PJI compared to aseptic loosening or primary arthroplasty. The area under the curve (AUC) in a receiver operating characteristic curve analysis was equal to 1.0 for both scores revealing excellent diagnostic accuracy. Isolated synoviocytes as cellular AMP source showed comparable results with a significant LL-37/HBD-3-increase up to 3 × in PJI. In contrast, local HBD-2 levels were negligible (*p* > 0.23) upon PJI with a lower diagnostic accuracy (AUC = 0.65) in analogy to our previous findings with synovial fluid.

**Conclusions:**

Our results implicate AMPs as promising and specific biomarkers for the histological diagnosis of PJI.

## Background

Total hip and knee joint replacement is one of the most successful surgical interventions worldwide. By now, very good to excellent long-term results exist. In consequence, implantation rates rise continuously. In the United States, for 2030, a two-time growth for THR and a five-time growth for TKR is estimated [1]. For Europe, an equivalent growth is anticipated with greater increase of THRs than TKRs [2]. In consequence, the number of septic revision arthroplasties rises as well and the economic burden of periprosthetic joint infection (PJI)-related surgical interventions grows considerably [1, 3, 4]. The incidence of PJI itself in primary arthroplasty rose from 1.99% in 2001 to 2.18% in 2009 to 1.5% to 4% nowadays [4, 5]. In revision procedures, an incidence of PJI up to 20% is reported [5]. The increase of morbidity, patient-specific risk factors and complexity of revision procedures are thought to be responsible [4].

For successful PJI therapy with implant retention or exchange arthroplasty, safe diagnosis with precise differentiation between septic and aseptic implant loosening is a mandatory prerequisite [2–4]. According to the Musculoskeletal Infection Society (MSIS) criteria, the “gold standard” of PJI diagnosis consists of a stepwise algorithmic, from non-invasive to more invasive measures [6, 7]. Diagnostic accuracy is high in acute PJI with typically purulent infection, severe illness and obvious signs of systemic inflammation (sepsis) [6, 8]. However, in the clinically more common chronic low-grade infections, diagnostic tools show insufficient accuracy [3, 8]. Patients present only with a chronically painful joint in the absence of systemic inflammatory response. Typically coagulase-negative *Staphylococci* (CoNS) with predominantly *Staphylococcus epidermidis* form resistant implant biofilms with high antibiotic resistance and reduced possibility of microbiological detection [3, 4, 9].

Innovative synovial biomarkers are favored to close this truly challenging diagnostic gap. They are the most accurate, rapid, cost-effective and least invasive tools available [10]. Among them antimicrobial peptides (AMPs) display the most promising candidates [10, 11]. AMPs are part of the innate immune response towards microbial growth. Due to their capacity to directly kill microbes at the site of infection they are considered the future of anti-infective prevention and therapy drugs [12, 13]. With their upregulation upon infection and direct antimicrobial activity, AMPs seem to be ideal for proofing locally acting low-grade PJI and at the same time ignoring general inflammatory conditions. Recently, we could show significant intraarticular levels with high diagnostic accuracy of the AMP human cathelicidin LL-37 and human β-defensin-3 (HBD-3) in PJI synovial fluid of hip and knee [14]. In contrast, AMP serum levels were unaltered upon infection. The AMP α-defensin is the first synovial fluid biomarker commercially available for specific PJI screening [15]. Indicating the high potential of AMPs, α-defensin outperforms current PJI diagnostics with excellent diagnostic accuracy, response to a wide spectrum of microbial organisms and resistance to prior antibiotic administration in limited studies [16–18].

However, compared to synovial fluid only very few publications exist suggesting the advantage of synovial membrane biomarkers for histopathological PJI diagnosis. In clinical routine, pathogenic (histological) diagnosis still displays one of the most powerful alternative local tools for diagnosing PJI as tissue can be harvested in 100% of cases intraoperatively compared to synovial fluid with its dependency on the amount of joint effusion. Especially in low-grade PJI, joint fluid aspiration can be challenging due to the typically low-grade synovialitis. The histopathological grading of periprosthetic infection membrane based on neutrophilic granulocytes count established by Morawietz et al. nearly more than 15 years ago still acts as important “gold standard” [19]. However, this unspecific histopathological grading just tells if there is an infection or not. Additional information about the type and timeline of infection, the underlying pathogen, etc. cannot be given. Regarding the promising AMPs, LL-37, β-defensin-2 (HBD)-2 and HBD-3 were not detectable in healthy human synovial membranes, but HBD-3 and LL-37 showed significant increase in pyogenic native arthritis [20]. Immunofluorescence staining of periprosthetic tissue stated to have more HBD-3-positive cells in PJI compared to aseptic loosening or native joints [21]. However, here information about the type of PJI (acute vs. low grade) and joints is missing, various coagulase-negative and coagulase-positive staphylococcal pathogens have been pooled. In another preliminary study, monocyte, macrophage and endothelial cells were assumed to be the major cellular sources of HBD-3 in the pseudocapsule/periprosthetic membrane [22]. However, the type of PJI is unclear, and a great variety of different pathogens has been pooled.

Consequently, the objective of the present study was to evaluate PJI synovial membrane and synoviocytes with CoNS as pathogens for the promising AMP biomarkers LL-37, HBD-3 and HBD-2 by easy-to perform semiquantitative immunohistochemical (IHC) analysis. This may significantly facilitate future histological diagnosis of PJI in clinical routine to improve outcome of artificial joint revision.

## Materials and methods

### Patients and study design

The study protocol was approved by the institutional review board of the Technical University of Munich (No. 2544/09). Informed consent was obtained from every patient prior to screening. For this prospective single-center controlled clinical trial (diagnostic level II) patients were consecutively enrolled and included into three groups. Group 1: primary arthroplasty (PA; *n* = 8): patients with osteoarthritis of the hip or knee joint undergoing primary total hip (THR) and knee (TKR) replacements. There was no history of previous joint infection or current systemic infection. Pre-, peri- and postoperative assessments (clinical condition, blood analysis, histological and 10-day microbial analysis of synovial fluid and synovial membrane) confirmed asepsis. Groups 2 and 3: surgical hip or knee arthroplasty revision due to aseptic loosening (AL; *n* = 9) or septic loosening with underlying PJI (SL; *n* = 8). For these two groups, synovial membranes from consecutive patients of our previous study who underwent revision of their THR or TKR complicated by aseptic loosening or PJI were used [14]. With these patients, we could show high diagnostic accuracy of the AMPs LL-37 and HBD-3 in PJI synovial fluid of hip and knee [14]. As described, implant loosening was assessed preoperatively by clinical features, radiographs, and, if necessary, bone scans. To differentiate between aseptic loosening and PJI, as per standard institutional procedure serum C-reactive protein (CRP), white blood cell count (WBC), erythrocyte sedimentation rate (ESR), joint aspirate leukocyte count and differential, microbiological analysis of joint aspirates and periprosthetic tissue samples (at least six samples, incubated aerobically and anaerobically for at least 10 days), and histopathological grading of periprosthetic synovial membrane according to the method of Morawietz et al. [19] were determined [14]. Compared to the inclusion of coagulase-negative (CoNS) and coagulase-positive staphylococcal pathogens in our previous study [14], the final diagnosis of PJI was confirmed in this study with the focus on the most common and challenging low-grade infections and their primary pathogens. Thus, combination of positive intraoperative microbiological culture with CoNS only and positive histopathological grading of infection was mandatory in all cases, serving as ‘‘gold standard’’ major criteria for the diagnosis of low-grade PJI according to the *Musculoskeletal Infection Society* based on [23]. Hereby possible differences in immunohistochemical analysis (IHC) among samples from patients with infections caused by a variety of bacterial pathogens should be minimized. Exclusion criteria for all groups were an autoimmune or other inflammatory disease, antibiotic treatment/previous infection within 2 months and surgical treatment within 3 months before surgery, partial joint replacement, and/or an allergy to implants, metal, or bone cement.

### Sample preparation (synovial membrane)

During surgery, joint aspirate and tissue biopsies for routine culture and histology were obtained. One additional biopsy of synovial membrane (inner surface of the joint capsule) closely related to the prosthesis was harvested and cut into halves. One half was immediately fixated (4% paraformaldehyde (PFA), 4 °C, 48 h) and dehydrated (ethanol). After washing with xylene paraffin-wax embedding of the synovial membrane was performed by running through several changes of paraffin (60 °C). Obtained tissue blocks were trimmed and inner layer (intima) of synovial membrane was sectioned (3 μm thickness) by a rotary microtome. With microscope slides, sections were picked out of the water bath and stored upright in a slide rack for drying (60 °C).

### Immunohistochemical analysis (synovial membrane)

After deparaffinization and xylene removal with 100% ethanol, slides were hydrated in a series of graded alcohols until water was used. For endogenous peroxidase blocking, sections were washed with phosphate buffer saline (PBS), 1% hydrogen peroxide (20 min) and PBS again [24]. Antigen-retrieval was performed by unmasking antigenic epitopes with proteinase K (for HBD-2 and LL-37) (Qiagen, Hilden, Germany) or microwave heating (for HBD-3) [24]. For IHC staining, as described by the manufacturer primary antibodies against human HBD-2 (source goat; R&D Systems, Wiesbaden, Germany), HBD-3 (source rabbit; Lifespan, Seattle, USA) or LL-37 (source rabbit; Innovagen, Lund, Sweden) were applied overnight followed by PBS washing (10 min). Then biotinylated secondary antibodies for IHC of HBD-2 (anti-goat: Vector Laboratories, Burlingame, USA), HBD-3 or LL-37 (anti-rabbit: Vector Laboratories, Burlingame, USA) and avidin–biotin complex (ABC) (Vector Laboratories, Burlingame, USA) were administered, each for 30 min. After sufficient red staining (approximately 5–8 min) with 3-amino-9-ethylcarbazole (AEC) (Dako, Hamburg, Germany), washing with PBS and nuclear counterstaining for contrast gain (2 min Mayer’s hämalaun solution), sections were sealed with glycerin–gelatin and a cover glass. Isotype control (goat: R&D Systems, Wiesbaden, Germany; rabbit: PeproTech, Rocky Hill, USA), negative control (omission of the primary antibodies) and positive control (skin samples of psoriasis known to exhibit high expression of HBD-2, HBD-3 and LL-37 in the epidermis [25]) were performed (data not shown).

### Sample preparation (synoviocytes)

The other half of the additional biopsy of synovial membrane (inner surface of the joint capsule) closely related to the prosthesis and harvested during surgery was used. Under microscope, the inner layer (intima) was separated and digested with Liberase. With nylon-net filters (70 μm and 40 μm) and centrifugation (1140 rpm, 4 °C, 10 min), isolated synoviocytes were seeded in cell culture medium. With trypan blue dye exclusion test, viable cells were counted and synoviocytes seeded at 4000 cells/cm^2^ in standard multiwell tissue culture plates. After cell culture amplification, trypsin-released synoviocytes from passage 3 (30 days) were placed on chamber slides (20,000 cells in 1 ml cell culture medium/chamber). After monolayer generation (2 days), cells were PBS washed, air dried and fixated (1:1 ethanol/acetone).

### Immunohistochemical analysis (synoviocytes)

After rehydration in PBS and protein block for 15 min (Dako, Hamburg, Germany), primary antibodies against human HBD-2, HBD-3 and LL-37 as described above, as well as primary antibodies against synoviocyte verification markers human CD 68 (source mouse; Dako, Hamburg, Germany), HSP 27 (source mouse; Santa Cruz, Heidelberg, Germany), Polylaminin (source rabbit; Dako, Hamburg, Germany) and Vimentin (source mouse; Dako, Hamburg, Germany) were applied overnight (first/second chamber). Isotype control (goat: R&D Systems, Wiesbaden, Germany; rabbit: PeproTech, Rocky Hill, USA and mouse: Dako, Hamburg, Germany) and negative control (omission of the primary antibodies) were placed in chambers 3 and 4 overnight. After PBS washing (10 min), biotinylated secondary antibodies (anti-goat, anti-rabbit and anti-mouse: Vector Laboratories, Burlingame, USA) and ABC (Vector Laboratories, Burlingame, USA) were administered as described above, each for 30 min. After sufficient red staining (approximately 5–8 min) with AEC (Dako, Hamburg, Germany), washing with PBS and nuclear counterstaining for contrast gain (2 min Mayer’s hämalaun solution), chamber slides were sealed with glycerin–gelatin and a cover glass. Positive control of AMPs was performed as described above [25], for synoviocyte verification, the well-known immortalized synoviocyte cell line K4IM was used and synoviocyte stains from the current literature were compared.

### Microscopy and semiquantitative staining analysis

Samples were visualized using the Axio Observer.Z1 (Carl Zeiss Microscopy GmbH, Jena, Germany) with × 10 magnification. 5 representative tissue slides (synovial membrane) and chamber slides (synoviocytes) per patient and antibody were evaluated, each with 3 representative field of views from different regions containing 100 single cells. All evaluations were performed twice by individual microscopical analysis of two independent experienced investigators according to the following two commonly used and numerous validated uniform scoring-systems. The Immune Reactive Score (IRS) described by Remmele and Stegner in 1987 [26] is based on multiplication of the number of stained cells with their staining intensity (SI). The number of stained cells is classified from 0 to 4 (0: 0% stained cells, 1: < 10% stained cells, 2: ≤ 50% stained cells, 3: 51–80% stained cells, 4: 81–100% stained cells), the staining intensity is classified into 4 groups (0: no color reaction, 1: weak staining, 2: moderate staining, 3: strong staining). With a possible final range from 0 to 12, IRS scores of 0–2 are considered negative, scores of 3–12 are considered positive [26]. The Total Score (TS) described by Allred in 1998 [27] adds the number of stained cells with their staining intensity. The number of stained cells is classified from 0 to 5 (0: 0% stained cells, 1: 0–1/100 stained cells, 2: 1/100–1/10 stained cells, 3: 1/10–1/3 stained cells, 4: 1/3–2/3 stained cells, 5: 2/3–3/3 stained cells), the staining intensity is classified into 4 groups (0: no color reaction, 1: weak staining, 2: moderate staining, 3: strong staining). With a possible final range from 0 to 8, TS scores of 0–2 are considered negative, scores of 3–8 are considered positive [27]. Comparing both scores the IRS emphasizes more the staining intensity, the TS accentuates more the number/percentage of stained cells. Both scores taken together interpret the IHC result most significant.

### Statistical analysis

The distribution of quantitative data is presented by mean and standard deviation. Hypothesis testing for group differences was performed by exact Wilcoxon rank-sum tests. Sample size was chosen as described [27]. Additionally for synovial membrane, diagnostic accuracy (discriminatory strength) of the AMPs for identification of PJI was determined as previously described for synovial fluid [13] on the basis of the area under the curve (AUC) value obtained from a receiver operating characteristic (ROC) curve analysis. An AUC of 0.5 indicates that a test has no diagnostic strength, and a test with an AUC of ≥ 0.9 (maximum possible value, 1.0) is considered to have excellent diagnostic strength = high sensitivity and high specificity. Exploratory hypothesis testing was performed on two-sided 5% significance levels. Statistical analyses were performed by our professional statistician and co-author with use of IBM SPSS Statistics (version 26.0, Armonk, NY, IBM Corp.) and R (version 3.6.3; R Foundation, Vienna, Austria).

## Results

For classification of THR and TKR patients into primary arthroplasty group (PA), surgical revision group due to aseptic loosening (AL) and septic arthroplasty group (SL with confirmed coagulase-negative staphylococcal infection) according to the criteria of the Musculoskeletal Infection Society (MSIS), amongst others preoperative blood serum inflammatory parameters WBC and CRP levels were compared (Table 1). WBC differences between all groups were not relevant (a; *p* values: PA vs. AL = 0.815, PA vs. SL = 0.901, AL vs. SL = 0.888). Additionally, CRP levels did not reach significance between AL and PA (b; *p* = 0.167). In contrast in SL CRP levels were significantly increased (c) up to 17× compared to AL (*p* = 0.007) and up to 25x compared to PA (*p* = 0.006).

**Table 1:** Preoperative blood serum inflammatory parameters: white blood cell count (WBC) and C-reactive protein (CRP)

Group	WBC(×10^9^)^a^	C-reactive Protein (mg/dl)^b^
Comparison of preoperative white blood cell count (WBC) and C-reactive protein in blood serum		
Primary arthroplasty	6.75 ± 1.37	0.33 ± 0.22^c^
Aseptic loosening	6.87 ± 1.63	0.48 ± 0.19^c^
Septic loosening	7.99 ± 3.75	8.10 ± 6.26

Comparison between any two groups revealed no significant difference in WBC (a). Regarding CRP no significant difference could be found between aseptic loosening and primary arthroplasty (b). However, septic loosening led to a significant CRP increase compared to aseptic loosening (17x) and primary arthroplasty (25×) (c)

^*a*^*p* >* 0.05 between all groups*

^*b*^*p* >* 0.05 between primary arthroplasty and aseptic loosening*

^*c*^*p* <* 0.05 in comparison to PJI loosening*

**Fig. 1:**
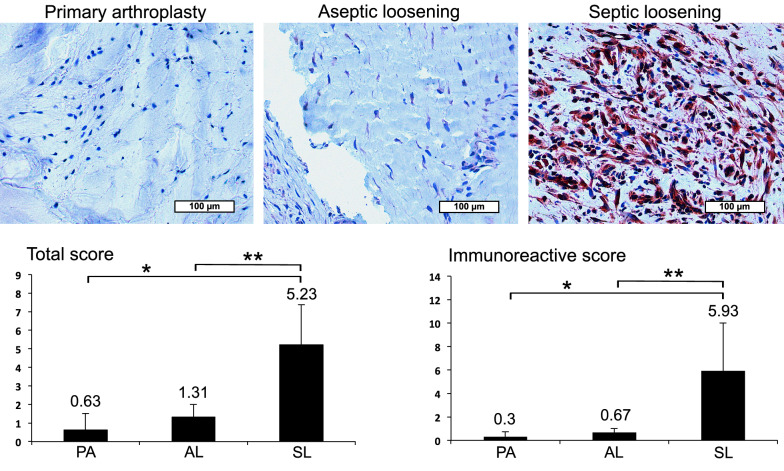
Immunohistochemical LL-37 analysis in PJI synovial membrane. Representative stained paraffin sections with enhanced dyeing of LL-37 in patients with septic endoprosthetic loosening (SL) compared to aseptic endoprosthetic loosening (AL) and primary arthroplasty (PA). Semiquantitative analysis revealing significant (*p* < 0.05) LL-37 increase in SL vs. AL (up to ×9; **) and PA (up to ×20; *)

**Fig. 2:**
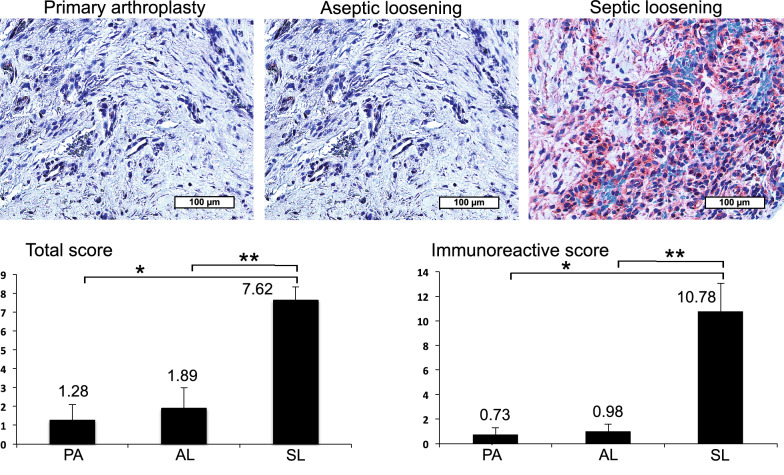
IHC HBD-3 analysis in PJI synovial membrane. Representative stained paraffin sections with enhanced dyeing of HBD-3 in patients with SL compared to AL and PA. Semiquantitative analysis indicating significant (*p* < 0.05) HBD-3 increase in SL vs. AL (up to ×11; **) and PA (up to ×15; *)

Then immunohistochemical analysis (IHC) of synovial membrane, the area of joint fluid production was performed with representative stained paraffin sections of patients with PA, AL and SL (Figs. 1, 2). With LL-37 a clearly visible enhanced red dyeing in patients with SL compared to AL and PA was obvious (Fig. 1). This could be confirmed by semiquantitative analysis revealing significant LL-37 increase up to 9x in SL vs. AL and up to 20x in SL vs. PA (Fig. 1). The area under the curve (AUC) in a receiver operating characteristic curve analysis was equal to 1.0 revealing excellent diagnostic accuracy for LL-37 in diagnosing PJI. In accordance, comparison of LL-37 levels in PA and AL revealed no relevant differences neither in visible dyeing nor in semiquantitative analysis. Comparable results were obtained with HBD-3 showing an obviously enhanced red dyeing in patients with SL compared to AL and PA (Fig. 2). Objective quantification indicated a significant HBD-3 increase up to 11x in SL vs. AL and up to 15x in SL vs. PA (Fig. 2). The AUC analysis was equal to 1.0 revealing also excellent diagnostic accuracy for HBD-3 in diagnosing PJI. Accordingly, comparison of PA and AL HBD-3 levels revealed no relevant difference neither in visible dyeing nor in semiquantitative analysis. In contrast HBD-2 visible dyeing as well as semiquantitative analysis showed only negligible differences between PA (Total Allred Score (TS) 0.3 and Immunoreactive Remmele and Stegner score (IRS) 0.15), AL (TS 1.2 and IRS 0.6) and SL (TS 1.38 and IRS 0.7). In accordance, the AUC value was 0.65 revealing a poor diagnostic accuracy.

**Fig. 3:**
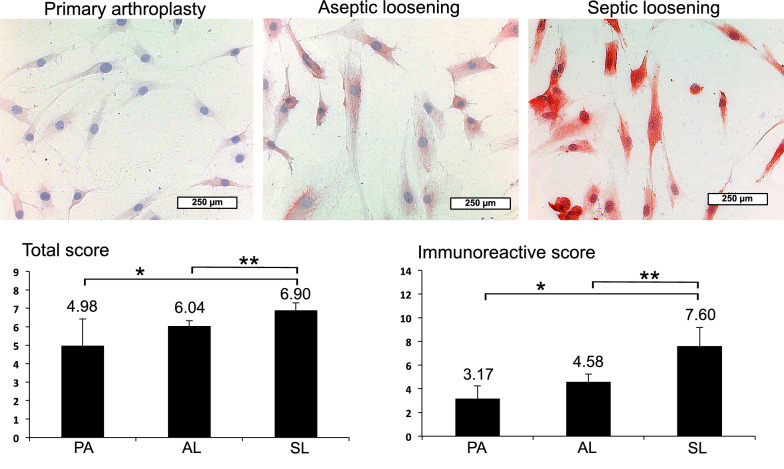
IHC LL-37 analysis of isolated synoviocytes from PJI synovial membrane. Representative stained slides with enhanced dyeing of LL-37 in synoviocytes from SL compared to AL and PA. Semiquantitative analysis revealing significant (*p* < 0.05) LL-37 increase in SL vs. AL (up to ×2; **) and PA (up to ×2; *)

**Fig. 4:**
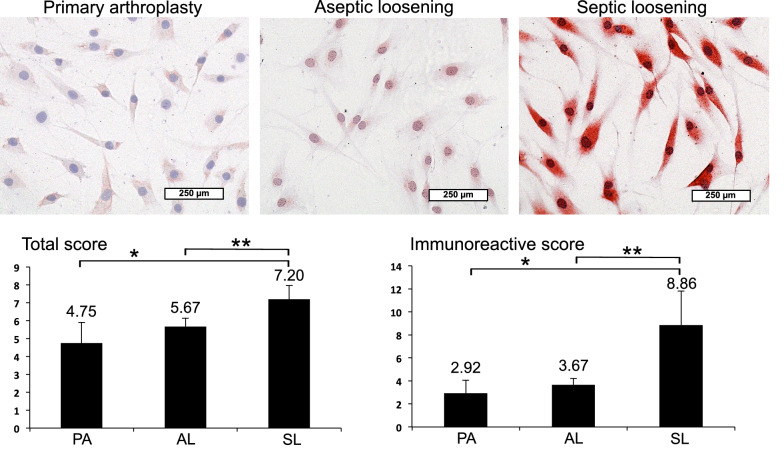
IHC HBD-3 analysis of isolated synoviocytes from PJI synovial membrane. Representative stained slides with enhanced dyeing of HBD-3 in synoviocytes from SL compared to AL and PA. Semiquantitative analysis revealing significant (*p* < 0.05) HBD-3 increase in SL vs. AL (up to ×2; **) and PA (up to ×3; *)

Finally, IHC of synoviocytes, the cells responsible for joint fluid production, was performed with representative stained slides (Figs. 3, 4). Synoviocytes were isolated from synovial membrane of the same above-mentioned patients with PA, AL and SL, confirmed by synoviocytal markers CD 68, HSP 27, Polylaminin and Vimentin and amplified ex vivo. Also, on the single cell level, LL-37 showed a visibly enhanced red dyeing in SL compared to AL and PA (Fig. 3). This could be confirmed by semiquantitative analysis revealing significant LL-37 increase up to 2x in each SL vs. AL and SL vs. PA (Fig. 3). Between PA and AL, only insignificant LL-37 levels could be found in visible dyeing and semiquantitative analysis. Similar results were obtained with HBD-3 showing an enhanced red dyeing in patients with SL compared to AL and PA (Fig. 4). Objective quantification indicated a significant HBD-3 increase up to 2x in SL vs. AL and up to 3x in SL vs. PA (Fig. 4). Again, differences of HBD-3 levels appeared to be negligible between PA and AL regarding visible dyeing and semiquantitative analysis. In contrast, HBD-2 showed no relevant changes in visible dyeing and semiquantitative analysis between PA (TS 4.25 and IRS 2.67), AL (TS 2.82 and IRS 1.72) and SL (TS 3.24 and IRS 2.02).

## Discussion

The worldwide continuously rising number of total hip and knee joint replacements goes along with a rising rate of septic and aseptic revision arthroplasties [1, 3, 4] and at the same time a rising incidence of PJI [5]. Despite consistent medical improvement, increase of morbidity, patient-specific risk factors and complexity of revision procedures are thought to be responsible [4]. Thus, safe diagnosis of PJI and its related complications (endoprosthetic loosening) is up-to-date more than ever. However, the major challenge in diagnosing PJI is still diagnostic uncertainty [5].

For acute PJI, diagnostic accuracy is high due to systemic inflammation, caused by highly virulent organisms such as coagulase-positive *Staphylococcus aureus* and to a lesser extinct beta-hemolytic streptococci and aerobic Gram-negative bacilli [6, 8]. Fast implant-preserving surgical revision with exchange of mobile parts is the gold standard with high success rates [5]. In contrast, for the more common chronic low-grade PJI, diagnostic tools still show insufficient accuracy due to an overall weak or even missing systemic inflammatory response [3, 8]. Underlying lower virulent pathogens are mostly coagulase-negative staphylococci (CoNS) as in this study with predominantly *Staphylococcus epidermidis* and to a lesser extinct *Propionibacterium acnes (P. acnes)*. Due to their formation of resistant implant biofilms with reduced possibility of microbiological detection and high antibiotic resistance, safe differentiation between chronic low-grade PJI and aseptic loosening is still challenging [3–5, 9]. Novel biomarkers are most promising to close this diagnostic gap to choose the correct treatment.

Systemic biomarkers such as WBC are, in contrast to other infections, generally not useful in safely diagnosing (low-grade) PJI as shown in this study and known from others [14, 21]. However, CRP still plays its role as the most common systemic marker for differentiation between PJI and aseptic loosening with a reported possible diagnostic accuracy up to 84% [28]. In accordance with this study and others, CRP blood levels can be elevated [14, 21]. However, interference with systemic diseases such as other infections, rheumatoid arthritis or gout lowers its specificity remarkably. On the other side, normal CRP values not exclude PJI, especially when caused by low virulence *P. acnes* and others. Thus, local biomarkers have been favored over the last decade for diagnosing PJI as most accurate, rapid, cost-effective and least invasive tools available [5, 29].

In synovial fluid, antimicrobial peptides (AMPs) display the most promising candidates due to their direct and specific local antimicrobial activity against the underlying pathogen [29]. We were the first to show significant levels of AMPs, human cathelicidin LL-37 and β-defensin-3 (HBD-3) with high diagnostic accuracy in PJI synovial fluid [14]. In the course also the synovial fluid AMP α-defensin has been proven to have excellent diagnostic accuracy in the detection of PJI up to a commercially available test since 2 years [29]. In multicenter studies, AMPs have been shown to outperform classical synovial fluid parameters such as WBC, CRP, percent segmented neutrophils and erythrocyte sedimentation rate [29, 30]. Their capacity for PJI detection has been shown to be independent of prior antibiotic treatment, synovial fluid blood contamination, immunosuppression or natural skin flora. Additionally, they respond to a wide spectrum of PJI organisms [29, 30]. However, based on the current evidence with only very few meta-analyses (laboratory-based tests and test kits), synovial biomarkers cannot be applied yet as a standalone diagnostic tool [30]. Additionally, especially in low-grade PJI synovial fluid can be difficult or even impossible to harvest due to typically low-grade synovialitis without associated joint effusion. Therefore, in clinical routine, pathogenic (histological) diagnosis still displays one of the most powerful alternative local tools for diagnosing PJI.

Tissue with suspected PJI is routinely collected during surgery for bacterial culture. However, bacterial culture is affected by various factors such as antibiotic pretreatment, number of samples, time limit, length of cultivation time and contamination possibility. As a result, false-positive or false-negative findings are a known problem, especially in low-grade PJI [5]. Thus, the histopathological grading of PJI membrane based on neutrophilic granulocytes count established by Morawietz et al. nearly more than 15 years ago still acts as a MSIS “gold standard” [19]. Besides its appreciated power in predicting PJI, further information of the type and timeline of infection and the underlying pathogen cannot be given due to its methodical non-specificity. However, compared to synovial fluid, only very few publications exist suggesting the advantage of synovial membrane biomarkers for histopathological PJI diagnosis. Knowing their advantages in synovial fluid, the promising AMPs could outperform bacterial culture and neutrophilic granulocytes count for the detection of PJI regarding diagnostic accuracy and user-friendliness. Furthermore, the pathogen-specific action of AMPs could outperform the current Morawietz et al. score by giving additional information of the type of infection and the underlying pathogen improving the treatment.

As known from Paulsen et al., by RT-PCR, the AMPs LL-37, β-defensin-2 (HBD)-2 and HBD-3 are not detectable in healthy human synovial membranes, but HBD-3 and LL-37 show significant increase in pyogenic native arthritis [20]. This is in accordance with our previous findings in PJI synovial fluid [14]. With the present study it could be confirmed in PJI synovial membrane (area of joint fluid production) as well. Significantly enhanced IHC staining of LL-37 and HBD-3 was already clearly visible in septic arthroplasty group (SL) compared to primary arthroplasty group (PA) and aseptic loosening (AL) and could be validated by semiquantitative analysis. Additionally with the present study, excellent diagnostic accuracy of LL-37 and HBD-3 for PJI, as known from synovial fluid [14], could be confirmed for synovial membrane as well. The fact that there were no relevant differences in AMP IHC staining between primary arthroplasty group (PA) and aseptic loosening (AL) favors AMPs as PJI tissue markers. IHC analysis of HBD-2 in synovial membrane showed only insignificant differences between the three groups revealing no relevant diagnostic accuracy in PJI. This is in accordance with our previous observations with synovial fluid [14]. Liu et al. stated to have more HBD-3-positive cells with immunofluorescence staining of periprosthetic tissue in PJI compared to aseptic loosening or native joints [21]. However, information about the type of PJI (acute vs. low grade) and joints is missing, various CoNS and coagulase-positive staphylococcal pathogens up to three cases with uncultured bacteria have been pooled. In contrast, in the present study, the focus was set on the clinically most relevant and highly challenging low-grade PJI by strict inclusion of CoNS only as their primary pathogens. Knowing the lower virulence of these pathogens, all PJI cases were additionally confirmed by positive gold-standard histopathological grading of infection (neutrophilic granulocyte count).

On the cellular level, Levon et al. assumed in a preliminary study monocyte, macrophage and endothelial cells to be the major cellular sources of HBD-3 in the pseudocapsule/periprosthetic membrane [22]. However, the type of PJI is unclear, again a great variety of different pathogens has been pooled. In contrast, in the present study, IHC analysis of isolated and validated synoviocytes from the same synovial membrane of the same above-mentioned patients with strict inclusion of underlying CoNS only in case of PJI was performed. Hereby our findings from synovial membrane could be confirmed by significantly enhanced IHC staining of synoviocytes as cellular source of LL-37 and HBD-3 production in SL compared to PA and AL. Again, the fact that there was no relevant difference in AMP IHC staining between PA and AL on the single cell level (responsible for joint fluid production) favors AMPs as PJI tissue markers.

The study has various limitations. We included only confirmed coagulase-negative staphylococcal infections as typical low-grade PJI pathogens to minimize the potential difference in antimicrobial peptide expression among samples from patients diagnosed with infections with a variety of bacterial species. Although this is a potential limitation of our results with regard to their utility in identifying cases of PJI caused by other bacteria, we believe that our study presents important and robust data and specifically addresses for the first time the clinically more common and challenging low-grade PJI. Another limitation is the relatively small sample size. Future studies are necessary to establish synovial biomarker analysis as a histological standard tool in the clinical setting elucidating the best cut-off values.

As previously described, AMPs serve predominantly as local antimicrobial agents, with a lesser systemic impact [31]. This hypothesis is confirmed now with synovial fluid of our previous study [14] and synovial membrane of the present study, both demonstrating significant local upregulation of LL-37 and HBD-3 in patients with PJI compared with aseptic loosening, whereas analysis of systemic levels failed to show significant differences between the groups [14].

## Conclusions

We conclude that IHC semiquantitative analysis of local antimicrobial peptide (AMP) expression in synovial membrane as well as synoviocytes provides valuable information for differentiating between periprosthetic aseptic and septic inflammatory processes. As known from joint fluid, the synovial LL-37 and HBD-3 showed high diagnostic accuracy in synovial membrane for distinguishing between aseptic and septic implant loosening. Further studies are needed to validate the potential of AMPs as promising specific histopathological tool in the diagnosis of PJI. With a desirable focus on the clinically challenging low-grade form, outcome of artificial joint revision should be improved.

## Data Availability

The datasets analyzed for the current study are available from the corresponding author on reasonable request.

## Abbreviations

AL: Aseptic endoprosthetic loosening
AMP: Antimicrobial peptide
AUC: Area under the curve
CoNS: Coagulase-negative staphylococci
CRP: C-reactive protein
ESR: Erythrocyte sedimentation rate
HBD-2: Human β-defensin-2
HBD-3: Human β-defensin-3
IHC: Immunohistochemical analysis
IRS: Immunoreactive Remmele and Stegner score
LL-37: Human cathelicidin
MSIS: Musculoskeletal Infection Society
PJI: Periprosthetic joint infection
PA: Primary arthroplasty
SL: Septic endoprosthetic loosening
THR: Total hip replacement
TKR: Total knee replacement
TS: Total Allred Score
WBC: White blood cell count
